# Effect of Sulfide and Chloride Ions on Pitting Corrosion of Type 316 Austenitic Stainless Steel in Groundwater Conditions Using Response Surface Methodology

**DOI:** 10.3390/ma17010178

**Published:** 2023-12-28

**Authors:** Jin-Seok Yoo, Nguyen Thuy Chung, Yun-Ho Lee, Yong-Won Kim, Jung-Gu Kim

**Affiliations:** Department of Materials Science and Engineering, Sungkyunkwan University, 2066, Seobu-Ro, Jangan-Gu, Suwon-Si 16419, Republic of Korea; wlstjr5619@skku.edu (J.-S.Y.); chung.ngthuy@g.skku.edu (N.T.C.); yunho0228@naver.com (Y.-H.L.); dyddnjs98@skku.edu (Y.-W.K.)

**Keywords:** spent nuclear fuel, disposal canister, stainless steel, response surface methodology, soil corrosion

## Abstract

This study investigates the corrosion resistance of Type 316 stainless steel as a candidate material for radioactive waste disposal canisters. The viability of stainless steel is examined under groundwater conditions with variations in pH, bisulfide ions (HS^−^), and chloride ions (Cl^−^) concentrations. Utilizing response surface methodology, correlations between corrosion factors and two crucial response variables, passive film breakdown potential and protection potential, are established. Cyclic potentiodynamic polarization tests and advanced analytical techniques provide detailed insights into the material’s behavior. This research goes beyond, deriving an equation through response surface methodology that elucidates the relationship between the factors and breakdown potential. HS^−^ weakens the passive film and reduces the pitting corrosion resistance of the stainless steel. However, this study highlights the inhibitory effect of HS^−^ on pitting corrosion when Cl^−^ concentrations are below 0.001 M and at equivalent concentrations of HS^−^. Under these conditions, immediate re-passivation occurs from the destroyed passive film to metal sulfides such as FeS_2_, MoS_2_, and MoS_3_. As a result, no hysteresis loop occurs in the cyclic polarization curve in these conditions. This research contributes to the understanding of Type 316 stainless-steel corrosion behavior, offering implications for the disposal of radioactive waste in geological repositories.

## 1. Introduction

Nuclear power stands out as a crucial energy resource in the battle against climate change due to its minimal carbon emissions. However, the disposal of radioactive spent nuclear energy, a byproduct of nuclear power generation, presents a formidable challenge. This waste, with half-lives extending from 10,000 to 1,000,000 years, demands secure storage deep underground with geologically stable conditions for prolonged periods [1,2]. Although disposal canisters for spent nuclear fuel are shielded by backfill materials and isolated from the soil environment, groundwater infiltration may occur after a long period of time [3]. Therefore, the canister materials need to be corrosion-resistant to withstand long-term exposure to the groundwater environment.

Numerous studies have identified that copper, Ni alloys, and Ti alloys are suitable materials for disposal canisters [4,5,6]. Notably, research in Sweden and Finland delves into copper canisters, while research in the United States has focused on Ni alloys and Ti alloys [7]. Although stainless steel is also a material with excellent corrosion resistance and high strength, it was evaluated as unsuitable for a spent nuclear fuel canister. This oversight is attributed to its vulnerability to localized corrosion in soil environments. Previous research has shown that sulfur, which originates from the oxidation of pyrite in the host rock and sulfate-reducing bacteria in soil environments, could enhance localized corrosion of austenitic stainless steels in the presence of chloride ions (Cl^−^) [7,8,9,10]. Furthermore, Lee et al. reported that hydrogen sulfide ions, so-called bisulfide ions (HS^−^) were found to generate defective and less resistive passive film on Type 316L stainless steel [11,12].

However, A. Elbiache and P. Marcus reported that molybdenum (Mo) could improve the resistance to sulfur of Fe-Cr-Ni-Mo alloys in sulfuric acid conditions [13]. Additionally, Akiko et al. demonstrated that Mo could prevent localized corrosion of Ni-Cr-Mo-Fe alloys by producing a molybdenum sulfide passive film with cation selectivity in the NaCl and H_2_S environments [14]. Recent research by Xiao et al. indicated that sulfur ions (S^2−^) enhanced the re-passivation ability of 2205 duplex stainless steel and prevented pitting corrosion in the NaCl solution with the high pH [15].

According to the SKB-TR-10-67 report, when the canister was closed and the bentonite used as backfill was saturated (up to 100 years, oxygen existed), the values of pH, HS^−^ and Cl^−^ in the groundwater were expected to be 6.8–8, 0–1.3 mM, and 0.05–0.25 M, respectively [16]. Therefore, unlike previous studies, this research aims to explore the corrosion behavior of Type 316 stainless steel and the effects of pH, HS^−^, and Cl^−^ under weak alkaline conditions with very low concentrations in groundwater.

To achieve this, response surface methodology (RSM) is used in this study. It is a highly efficient statistical analysis method that is useful for finding the relationship between several factors and one or more responses with a reasonable number of experiment designs [17]. Additionally, RSM can be used to generate a meaningful quadratic model to help predict the localized corrosion of stainless steel. In this study, RSM was employed to discern the relationship between the three corrosion factors (pH, HS^−^, and Cl^−^) and two response variables: the passive film breakdown potential (E_break_) and the protection potential (E_prot_) of stainless-steel Type 316 (UNS S31600) [18,19,20]. Cyclic Potentiodynamic Polarization tests (CPP) were utilized to obtain the two response variables, and the experiment with the three factors was designed using the Box-Behnken design (BBD) to model E_break_ and E_prot_. Additionally, X-ray photoelectron spectroscopy (XPS) and cyclic voltammetry (CV) were conducted to analyze the passivation film concerning the corrosion factors. 

## 2. Materials and Methods

### 2.1. Specimen Preparation

In this study, commercial-grade stainless steel Type 316, rolled to a thickness of 500 μm, was utilized. The chemical composition of the specimens is presented in Table 1. Before all electrochemical testing, the specimens underwent polishing with Si-C paper ranging from grit 220 to 600 [21]. This step ensured uniformity among the specimens, establishing a standardized surface for testing. The exposed surface area of each specimen was precisely adjusted to 1 cm^2^ using silicon paste.

### 2.2. Design of Experiments for Response Surface Methodology

RSM, a modern statistical and mathematical technique, was employed to model the response variables influenced by multiple factors. This study aimed to investigate how three factors, namely pH (A), log[HS^−^] (B), and log[Cl^−^] (C), influence the response variables E_break_ and E_prot_ under groundwater conditions. The BBD was chosen for the experiment matrix design due to its efficiency when dealing with three factors. Minitab (Minitab 19, Minitab, LLC., State College, PA, USA) and Statistita (Statistita v10, Stasista, Hamburg, Germany) software were used for the design of experiments and statistical analysis [22]. 

**Figure 1:**
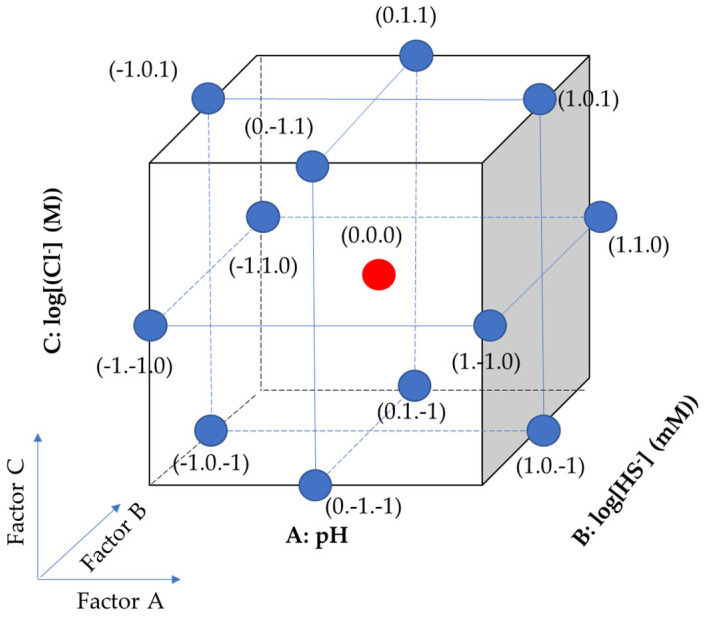
Schematic of BBD using this study.

To ensure a comprehensive investigation, this study focused on three factors: pH and concentrations of HS^−^ and Cl^−^. Table 2 outlines the ranges of pH and concentrations of HS^−^ and Cl^−^ examined in this study [23]. To simplify the adjustment and accuracy of the solutions, concentrations of HS^−^ and Cl^−^ were converted to a logarithmic scale. For statistical calculations, variables were coded using the minimum, medium, and maximum points of each variable separately, with values of −1, 0, and 1, respectively. The number of experiments was determined using the formula N = k^2^ + k + c_p_, where k represents the number of factors, 3, and c_p_ represents the number of experiments running at the center point, 3 [24]. The schematic of the design of experiments is illustrated in Figure 1 and Table 3, including 3 repeated center points and 12 different points [17]. Therefore, the total number of experiments to be conducted (N) was 15. Then, through the BBD results, a quadratic regression model can be created as shown in Equation (1).

(1) $$Y=\beta_{0\;}+\sum_{i=1}^{n}\beta_{i}X_{i}+\sum_{i=1}^{n}\beta_{\mathrm{ii}}X_{i}^{2}+\sum_{\begin{array}{c} i,j=1\\ i\neq j \end{array}}^{n}\beta_{\mathrm{ij}}X_{i}X_{j}$$

where Y is the response, β_0_ is a constant coefficient, β_i_, β_ii_, and β_ij_ are linear, quadratic, and interaction coefficients, respectively, and X_i_ and X_j_ are the coded values of the variables [24]. Responses were set to E_break_ and E_prot_, and the model quality was confirmed by analysis of variance (ANOVA).

### 2.3. Electrochemical Tests

Electrochemical tests, including CPP and CV, were conducted using a three-electrode method in a 1000 mL Pyrex glass cell. The specimens were connected to the working electrode, while a saturated calomel electrode (SCE) served as the reference electrode, and two glassy carbons were employed as the counter electrode. The experimental medium was distilled water, with HS^−^ concentration controlled using Na_2_S and Cl^−^ concentration controlled with NaCl. pH was adjusted using a 1 M sodium hydroxide solution and a saturated boric acid solution. All electrochemical tests were conducted using a VSP-300 instrument (Bio-Logic SAS, Seyssinet-Pariset, France) at 23 °C. The experiments included an open-circuit potential (OCP) for 24 h, and CPP scans started from 0 V vs. OCP with a rate of 0.166 mV/s. The back scan was initiated when the current density reached 0.1 mA/cm^2^ at the same rate. Surface images of all specimens were collected for comparative analysis using an optical microscope (OM), and for the 0.001 M Cl^−^ conditions, additional pitting information was collected using a confocal laser scanning microscope (CLSM). CV tests were also employed with OCP for 24 h, and the scan was repeated from −1.7 V_SCE_ to 0.7 V_SCE_ with a rate of 50 mV/s for 30 cycles. The solutions used for CV were pH 8, 1 mM HS^−^, and 0.1 M Cl^−^ and pH 8, 0.01 mM HS^−^, and 0.1 M Cl^−^.

### 2.4. XPS Analysis

XPS analysis was performed to analyze the chemical composition of the passive film after immersion for 24 h in different concentrations of HS^−^. The solutions used for XPS analysis were also the same with CV tests (pH 8, 1 mM HS^−^, and 0.1 M Cl^−^ and pH 8, 0.01 mM HS^−^, and 0.1 M Cl^−^). XPS analysis was carried out using an ESCA system (AXIS SUPRA, Kratos, Manchester, UK) with a monochromatic Al Kα radiation source (photoelectron energy of 1486.6 eV). High-resolution spectra were recorded in increments of 0.1 eV with a pass energy of 20 eV. The binding energies of the elements were calibrated to the carbon 1 s orbital at 284.8 eV.

## 3. Results and Discussion

### 3.1. Cyclic Polarization Measurements

To accurately determine the type of sulfur present in the solution, an understanding of the dissociation of H_2_S in aqueous solutions is essential. The reactions and equilibrium equations in the solution are as follows [11,15]:

(2) $$H_{2}S{=\mathrm{HS}}^{-}+H^{+},\;K_{a1}$$

(3) $${\mathrm{HS}}^{-}=S^{2-}+H^{+},\;K_{a2}$$

(4) CNa2S=CH2S×(1+Ka1CH+ +Ka1×Ka2CH+2)

Sun et al. suggested approximate values of *K_a_*_1_ and *K_a_*_2_ for Equations (2) and (3) at room temperature to be 10^−7^ mol/L and 10^−12^–10^−19^ mol/L, respectively [25]. Considering the pH range of 8–10 in this study, the concentration of H_2_S can be negligible because it is approximately 11 to 10^3^ times lower than the concentration of Na_2_S according to Equation (4), and the dissociation of HS^−^ is also negligible because of very low *K_a2_*. Thus, it was assumed that the concentration of HS^−^ used in this study was equivalent to the concentration of dissolved Na_2_S.

**Figure 2:**
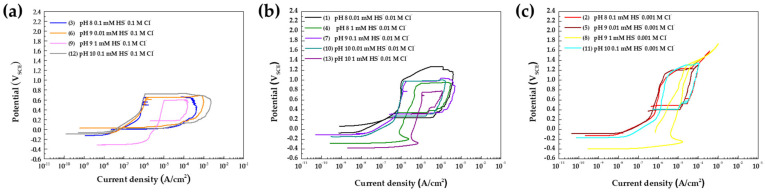
Cyclic polarization curves of Type 316 in the BBD according to the concentration of Cl^−^ (**a**) 0.1 M, (**b**) 0.01 M, and (**c**) 0.1 M.

Figure 2a–c depict the cyclic polarization curves with the designed experiment BBD according to the concentration of Cl^−^ and the parameters of the results of CPP are presented in Table 4. The surfaces of the specimens after CPP were measured using OM and CLSM, as shown in Figures S1 and S2. An important point in surface observation after CPP is that no pitting was found under No. 8 experiments (pH 9, HS^−^ 1 mM, Cl^−^ 0.001 M).

E_break_ values were selected at the point where the current density sharply increased within the passive region (<1 V_SCE_), while E_break_ reaching the transpassive region (>1 V_SCE_) was considered the point where the anodic curve’s slope changed. The current density at the E_break_ was set to the passive current density (i_pass_). Additionally, during the back scan, the point where the polarization curve intersects was defined as E_prot_. Regression models for E_break_ and E_prot_ were created and analyzed through ANOVA based on the results in Table 4.

### 3.2. Quadratic Regression Model and Analysis of Variance

The response variable, E_break_, was analyzed using Minitab 19 software to obtain a quadratic regression model as shown below:

(5) $$E_{\mathrm{break}\;}\left({\mathrm{mV}}_{\mathrm{SCE}}\right)=-1200+358\cdot \mathrm{pH}+139\cdot \log\left[{\mathrm{HS}}^{-}\right]-389\cdot \log\left[{\mathrm{Cl}}^{-}\right]-22.6\cdot {\mathrm{pH}}^{2}-75.3\cdot (\log{\left[{\mathrm{HS}}^{-}\right])}^{2}\;-14.8\cdot (\log{\left[{\mathrm{Cl}}^{-}\right])}^{2}-42.2\cdot \mathrm{pH}\cdot \log\left[{\mathrm{HS}}^{-}\right]+0.2\cdot \mathrm{pH}\cdot \log\left[{\mathrm{Cl}}^{-}\right]-27.5\cdot \log[{\mathrm{HS}}^{-}]\cdot \log[{\mathrm{Cl}}^{-}]$$

The statistical significance of Equation (5) should be assessed through the F-test and *p*-value [26]. The ANOVA results and coefficient of determination (R^2^) are presented in Table 5. ANOVA was employed to examine the relationship between the response variables and predictor variables. Generally, the significance level (α) was set to 0.05, and if the *p*-value of the regression model is less than α, it suggests that the model is statistically significant. In this model, the obtained F-value from the F-test was 15.39, exceeding the traditional threshold of F_critical_ (F_(9, 5, 0.05)_ = 4.77), and the *p*-value was below 0.05. Therefore, the model can reject the null hypothesis, which means that all coefficients are zero ($\beta_{1}=\beta_{2}=\beta_{3}=\beta_{11}=\beta_{22}={\;\beta }_{33}=\beta_{12}={\;\beta }_{13}=\beta_{23}=0)$ and the model is statistically significant. However, only variables with T-values outside the range of T_critical_ (T_(5, 0.05)_ = 2.75) are meaningful, and the results in Table 5 reveal that only the variable log[Cl^−^] (C) is significant in this model. Additionally, the R^2^ (pred.) value is only 48.54%, indicating that the model has an overfitting problem.

To address non-significant variables, a systematic revision of the model was conducted by eliminating each non-significant variable. The detailed R^2^ of revised Equations (5.1) and (5.6) summaries are provided in Table S1 of the supplementary section. The optimized Equation (6) was selected for the best model of E_break_ of the specimen.

(6) $$E_{\mathrm{break}\;}\left({\mathrm{mV}}_{\mathrm{SCE}}\right)=288.8-180.9\cdot \log\left[{\mathrm{HS}}^{-}\right]-300.1\cdot \log\left[{\mathrm{Cl}}^{-}\right]-72.7\cdot (\log{\left[{\mathrm{HS}}^{-}\right])}^{2}$$

With information from the optimized Equation (6) in Table 6, it is evident that the model has been significantly simplified and improved. The F-value is notably higher than F_critical_ (F_(3, 11, 0.05)_ = 3.59). Importantly, only one variable (B) is non-significant in this refined model, according to the T-test. Notably, R^2^ (pred.) exceeds 90%, while both R^2^ and R^2^ (adj.) remain higher than 90%, indicating a substantial improvement over the original model, and the model no longer exhibits signs of overfitting as observed previously.

**Figure 3:**
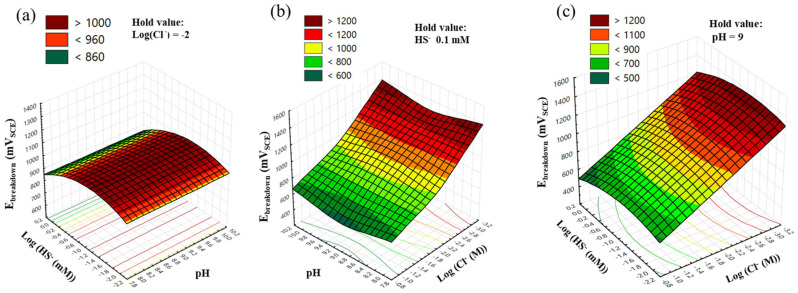
Response surfaces and contour plots from Equation (6) show the effect of (**a**) pH and log[HS^−^], (**b**) pH and log[Cl^−^], and (**c**) log[HS^−^] and log[Cl^−^] on the E_break_ of Type 316 stainless steel.

Figure 3a–c are response surfaces and contour lines from Equation (6). According to Equation (6) and Figure 3, E_break_ is entirely independent of pH and depends solely on Cl^−^ and HS^−^. This result affirms that the neutral-to-slightly basic pH of groundwater environments does not affect E_break_, aligning with previous studies [27,28]. Additionally, this model bears a striking resemblance to Strehblow and Titze’s Equation (7), illustrating the relationship between E_break_ and aggressive ion concentrations [29].

(7) $$E_{\mathrm{break}\;}\left({\mathrm{mV}}_{\mathrm{SCE}}\right)=A-B\cdot \log C_{\mathrm{agg}}$$

In Equation (7), A and B are constants, where B signifies the aggressiveness of the specific ions. In our study, Equation (6) exhibits similarities but introduces an additional quadratic term for HS^−^. This implies that the relationship with log[HS^−^] is not purely a negative linear effect, as observed with log[Cl^−^] under the given conditions. The E_break_ attains its maximum value when the concentration of HS^−^ is 0.058 mM (10^−1.24^ mM), as depicted in Figure 3c. However, given that the concentration of HS^−^ in groundwater is significantly lower than the concentration of Cl^−^, its influence appears unclear due to the dominating effect of Cl^−^. Particularly when the concentration of Cl^−^ is 0.1 M, confirming the effect of HS^−^ on localized corrosion of the specimen becomes challenging, as illustrated in Figure 2a.

**Figure 4:**
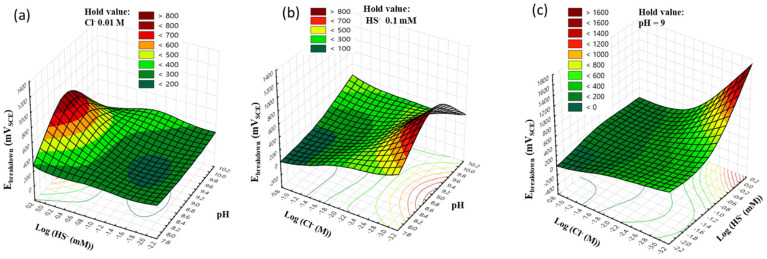
Response surfaces and contour plots from Equation (8) show the effect of (**a**) pH and log[HS^−^], (**b**) pH and log[Cl^−^], and (**c**) log[HS^−^] and log[Cl^−^] on the E_prot_ of Type 316 stainless steel.

The impact of HS^−^ is evident in E_prot_, which increased with a concentration of 1 mM HS^−^ at all Cl^−^ concentrations, as shown in Figure 4a–c and Table 4. To statistically analyze the change in E_prot_ by each factor, a regression model of E_prot_ was created. The model for E_prot_ is presented in Equation (8) and Table 7.

(8) $$E_{\mathrm{prot}}\left({\mathrm{mV}}_{\mathrm{SCE}}\right)=-8277+1832\cdot \mathrm{pH}+182\cdot \log\left[{\mathrm{HS}}^{-}\right]-226\cdot \log\left[{\mathrm{Cl}}^{-}\right]-102.7\cdot {\mathrm{pH}}^{2}+132.0\cdot (\log{\left[{\mathrm{HS}}^{-}\right])}^{2}\;+72.8\cdot (\log{\left[{\mathrm{Cl}}^{-}\right])}^{2}-15.0\cdot \mathrm{pH}\cdot \log\left[{\mathrm{HS}}^{-}\right]+5.2\cdot \mathrm{pH}\cdot \log\left[{\mathrm{Cl}}^{-}\right]-182.5\cdot \log[{\mathrm{HS}}^{-}]\cdot \log[{\mathrm{Cl}}^{-}]$$

However, as listed in Table 7, the F-value in Equation (8) was lower than F_critical_, while R^2^ (pred.) was also 0, indicating that this model was not statistically significant and was under an overfitting problem. Despite attempts to optimize the model depicted in Table S2, this issue persisted. The overfitting can be attributed to the significant deviation observed in experiment No. 8. In this experiment, with the same Cl^−^ concentration of 0.001 M (equivalent to 1 mM) and HS^−^ concentration, the polarization curve of the stainless steel did not even exhibit a hysteresis loop, suggesting that the specimen resisted pitting corrosion under these conditions. These results emphasize that HS^−^ is not exclusively an aggressive ion; it can act as an inhibitor, contributing to an increase in the resistance to pitting corrosion of the specimen, particularly when considering the ratio of HS^−^ concentration in the Cl^−^ condition.

### 3.3. Surface Analysis and Corrosion Mechanism

**Figure 5:**
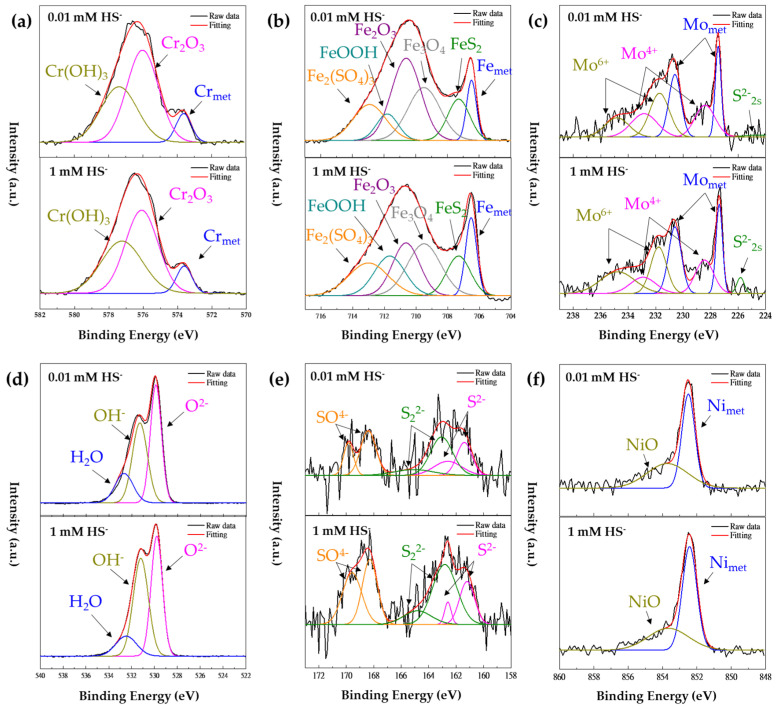
High-resolution scanning XPS spectra in different concentrations of HS^−^ (**a**) Cr 2p, (**b**) Fe 2p, (**c**) Mo 3d, (**d**) O 1s, (**e**) S 2p, and (**f**) Ni 2p.

XPS analysis was performed to provide evidence of surface changes and clarify the effect on the passive film depending on the concentration of HS^−^. Type 316 stainless steel is an Fe-Cr-Ni-Mo alloy, and Fe, Cr, and Mo can mainly affect the properties of the passivation film. The specimens used in the XPS analysis were immersed in 0.01 mM and 1 mM HS^−^ solutions for 24 h, with a fixed pH 8 and Cl^−^ concentration of 0.1 M, respectively. Figure 5a–f show the results of the XPS spectra of Cr 2p, Fe 2p, Mo 3d, O 1p, S 2s, 2p, and Ni 2p, respectively. Table 8 shows the binding energies of the primary compounds of the passive film obtained from XPS spectra deconvolution. The XPS spectra of Type 316 stainless steel showed no significant difference in its chemical compositions, even when the HS^−^ concentration changed. However, as the concentration of HS^−^ increased, the area fraction ratios of Cr(OH)_3_/Cr_2_O_3_ and OH^−^/O^2−^ increased, as shown in Table 9. Compared to Cr_2_O_3_, the content of Cr(OH)_3_ in the passive film has less protective resistance and more defects [30,31]. Therefore, when the concentration of HS^−^ increases, the protection of the passivation film decreases, so that i_pass_ increases, as shown in Figure 2 and Table 4. On the other hand, when HS^−^ is present in the solution, Ni is not significantly affected, but Fe and Mo sulfides, such as FeS_2_, MoS_2_, and MoS_3_, can be generated and act as a passive film, as shown in Figure 5b,c,e [32,33,34]. These results can also be confirmed through the Pourbaix diagram. The Pourbaix diagram of Fe-H_2_O-S and Mo-H_2_O-S systems presented by Davoodi et al. showed that FeS_2_ and MoS_2_ are stable phases in OCP measured in this study [35].

**Figure 6:**
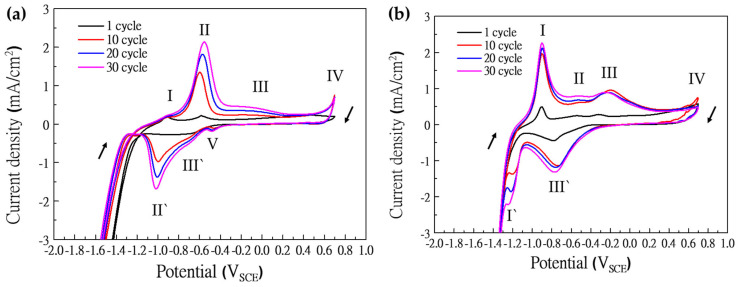
Results of CV according to concentration of HS^−^: (**a**) 0.01 mM, and (**b**) 1 mM.

Additionally, CV tests were performed for passive film analysis according to concentrations of HS^−^, as depicted in Figure 6a,b. When CV was performed depending on the concentration of HS^−^, a notable difference occurred in the oxidation peaks. Also, as the CV cycles were repeated, it was confirmed that the intensity of the peaks became more clear due to the metal ions eluted from the specimen. The peak I between −0.9 and −1.0 V_SCE_ indicates that Fe is oxidized to a Fe^2+^ ion, and the reduction peak of the reaction is displayed by peak I′. The pick II between −0.5 and −0.6 V_SCE_ indicates that the Fe^2+^ ion is oxidized to the Fe^3+^ ion, and the reduction peak of the reaction is represented by peak II′ [49]. The peak IV indicates that the Cr^3+^ ion is oxidized to the Cr^6+^ ion, and peak V indicates the cathodic reduction of dissolved oxygen [50]. The response of peak I depending on the concentration of HS^−^ is explained by the following equations [51].

(9) $$\mathrm{Fe}+HS^{-}\text{→}\;\mathrm{Fe}(\mathrm{HS}{)}_{\mathrm{ads}}+e^{-}$$

(10) $$\mathrm{Fe}(\mathrm{HS}{)}_{\mathrm{ads}}+{\mathrm{OH}}^{-}\text{→}\mathrm{FeS}+H_{2}O+e^{-}$$

Then, Haleem et al. suggested that peak III (~0.189 V_SCE_) also generated FeS_2_ with the following equation [52].

(11) $$\mathrm{Fe}+2HS^{-}+2OH^{-}\text{→}\mathrm{Fe}S_{2}+{2H}_{2}O+4e^{-}$$

On the other hand, other studies suggested that the peaks III and III′ are the reactions of MoS_4_^2−^ ions with the following equations [53,54].

(12) $$\mathrm{Mo}S_{4}^{2-}\text{→}\;\mathrm{Mo}S_{3}+\frac{1}{8}S_{8\;}+2e^{-}$$

(13) $$\mathrm{Mo}S_{3}+\frac{1}{8}S_{8}+2e^{-}\text{→}\mathrm{Mo}S_{4}^{2-}$$

(14) $$\mathrm{Mo}S_{4}^{2-}+2H_{2}O+2e^{-}\text{→}\;\mathrm{Mo}S_{2}+2HS^{-}+2OH^{-}$$

In peak III, MoS_3_ is generated by Equation (12). On the other hand, the peak III′ overlaps the reduction reactions of Equations (13) and (14). First, the reduction reaction of MoS_3_ occurs according to Equation (13), and then MoS_2_, a new reductive deposition material, is generated according to Equation (14) below about −0.55 V_SCE_.

**Figure 7:**
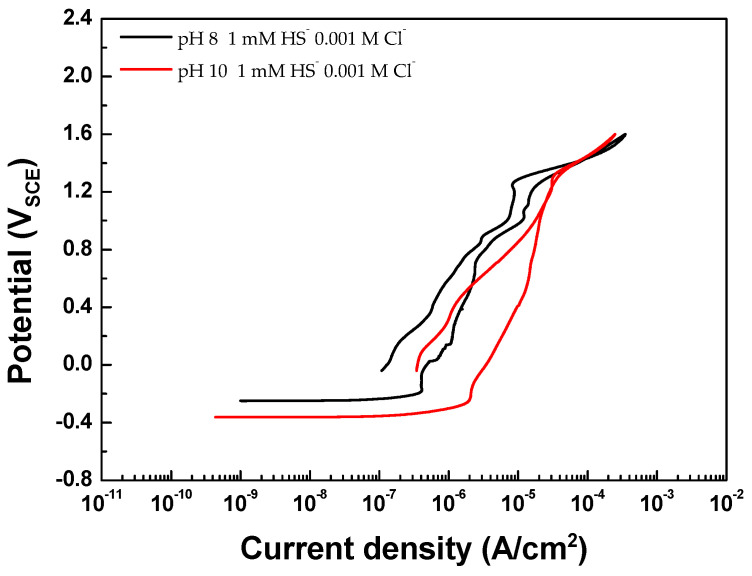
CPP curves according to pH 8 and 10 under 1 mM HS^−^ and 0.001 M Cl^−^ conditions.

Through XPS and CV analyses, it is clear that a new passive film is formed on the surface due to HS^−^. The presence of HS^−^ leads to an increased ratio of OH^−^/O^2−^, causing the dense and stable Cr_2_O_3_ on the passive film to decrease and become more susceptible to destruction. However, conversely, the broken passive film on the surface is re-passivated into new materials such as FeS_2_, MoS_2_, and MoS_3_ by the abundant HS^−^. Particularly when the concentrations of Cl^−^ and HS^−^ were equal in the groundwater condition, it was confirmed that the destroyed passivation film immediately underwent re-passivation, providing resistance to the corrosion of Type 316 stainless steel. Additionally, as depicted in Figure 7, no hysteresis loop was observed in the specimens after conducting CPP under conditions of pH 8 and 10, 0.001 M Cl^−^, and 1 mM HS^−^. This indicates that the resistance to pitting corrosion of Type 316 stainless steel remains unaffected by pH under these specific mildly alkaline conditions.

## 4. Conclusions

This study aimed to assess the suitability of Type 316 stainless steel for spent nuclear fuel disposal canisters, employing statistical analysis to scrutinize the impact of pH, HS^−^, and Cl^−^ on localized corrosion in groundwater. Key findings include:

- The concentrations of Cl^−^ and HS^−^ were significant factors affecting E_break_ on a logarithmic scale. Especially HS^−^ reduces the ratio of Cr_2_O_3_, which plays a crucial role in the passive film of stainless steel.
- On the other hand, HS^−^ reacts with Fe and Mo, the main components of the passive film on Type 316 stainless steel, forming new metal sulfides on the passive film and contributing to the re-passivation behavior of Type 316 stainless steel.
- Under specific conditions (Cl^−^ concentration 0.001 M, equal HS^−^ concentration), the alloy re-passivates the broken film immediately with metal sulfides (FeS_2_, MoS_2_, MoS_3_), displaying resistance to pitting corrosion.
- Therefore, regardless of pH, Type 316 stainless steel exhibits no hysteresis loop in the cyclic polarization curve under these conditions, suggesting negligible localized corrosion concerns.

In conclusion, this study identifies critical Cl^−^ and HS^−^ concentrations, affirming Type 316 stainless steel’s pitting resistance in weak alkaline groundwater with oxygen and holding valuable implications for its use in the disposal of radioactive waste in geological repositories.

## Figures and Tables

**Table 1:** Chemical composition of Type 316 stainless steel (wt.%).

Specimens		Composition (wt.%)								
	C	Cr	Ni	Mo	Mn	P	S	Si	N	Fe
Type 316	0.02	16.5	10.2	2.2	1.4	0.02	0.001	0.5	0.04	Bal.

**Table 2:** The investigated value range of factors in this study.

Investigated Factor	A, pH	B, log[HS^−^ (mM)]	C, log[Cl^−^ (M)]
Investigated range	8 to 10	−2 to 0	−3 to −1

**Table 3:** Experiments are designed based on BBD.

Standard Run	Coded Parameter			Real Parameter		
	A	B	C	pH	HS^−^	Cl^−^
					(mM)	(M)
1	−1	−1	0	8	0.01	0.01
2	−1	0	−1	8	0.1	0.001
3	−1	0	1	8	0.1	0.1
4	−1	1	0	8	1	0.01
5	0	−1	−1	9	0.01	0.001
6	0	−1	1	9	0.01	0.1
7	0	0	0	9	0.1	0.01
8	0	1	−1	9	1	0.001
9	0	1	1	9	1	0.1
10	1	−1	0	10	0.01	0.01
11	1	0	−1	10	0.1	0.001
12	1	0	1	10	0.1	0.1
13	1	1	0	10	1	0.01
14	0	0	0	9	0.1	0.01
15	0	0	0	9	0.1	0.01

**Table 4:** The results of the CPP are based on BBD.

Run	pH	HS^−^ (mM)	Cl^−^ (M)	E_OCP_ (mV_SCE_)	i_pass_ (μA/cm^2^)	E_break_ (mV_SCE_)	E_prot_ (mV_SCE_)
1	8	0.01	0.01	−64	1.14	999	227
2	8	0.1	0.001	−126	12.96	1238	483
3	8	0.1	0.1	−121	0.99	652	42
4	8	1	0.01	−313	10.19	942	339
5	9	0.01	0.001	−79	5.09	1209	389
6	9	0.01	0.1	29	1.32	649	33
7	9	0.1	0.01	−86	1.79	982	312
8	9	1	0.001	−398	39.6	1263	1263
9	9	1	0.1	−313	10.23	593	177
10	10	0.01	0.01	−144	1.33	984	271
11	10	0.1	0.001	−168	24.22	1310	409
12	10	0.1	0.1	−83	1.14	725	−11
13	10	1	0.01	−373	11.03	758	323
14	9	0.1	0.01	−108	2.14	1022	213
15	9	0.1	0.01	−176	2.53	1052	257

**Table 5:** Results of the ANOVA of Equation (5) for predicting E_break_.

ANOVA F_critical_ (DF_regression_, DF_residual_, α) (9, 5, 0.05) = 4.77					
Source	Degree of Freedom (DF)	Adj. Sum of Square	Adj. Mean Square	F-Value	*p*-Value
Regression	9	763,514	84,835	15.39	0.004
Residual	5	27,564	5513		
Lack of fit	3	25,097	8366		
Pure error	2	2467	1233		
Total	14	791,078			
Coded Coefficient					
T_critical_ (DF_residual_, α) (5, 0.05) = 2.57					
Term	Coefficient	Standard Error Coefficient	T-value	*p*-value	Remark
Constant	1018.7	42.9	23.76	0.000	<0.05
A	−6.7	26.3	−0.26	0.807	
B	−35.6	26.3	−1.36	0.233	
C	−300.1	26.3	−11.43	0.000	<0.05
AA	−22.6	38.6	−0.58	0.584	
BB	−75.3	38.6	−1.95	0.109	
CC	−14.8	38.6	−0.38	0.717	
AB	−42.2	37.1	−1.14	0.307	
AC	0.2	37.1	0.01	0.995	
BC	−27.5	37.1	−0.74	0.492	
Summary Model					
R^2^ = 96.52%		R^2^ (adj.) = 90.24%		R^2^ (pred.) = 48.54%	

**Table 6:** Results of the ANOVA of the revised Equation (6) for predicting E_break_.

ANOVA F_critical_ (DF_regression_, DF_residual_, α) (3, 11, 0.05) = 3.59					
Source	Degree of Freedom (DF)	Adj. Sum of Square	Adj. Mean Square	F-Value	*p*-Value
Regression	3	750,464	250,155	67.75	0.000
Residual	11	40,614	19,710		
Lack of fit	9	38,147	4239		
Pure error	2	2467	1233		
Total	14	791,078			
Coded Coefficient					
T_critical_ (DF_residual_, α) (11, 0.05) = 2.20					
Term	Coefficient	Standard Error Coefficient	T-value	*p*-value	Remark
Constant	997.3	24.6	40.57	0.000	<0.05
B	−35.6	21.5	−1.66	0.125	
C	−300.1	21.5	−13.97	0.000	<0.05
BB	−72.7	33.7	−2.31	0.041	<0.05
Summary Model					
R^2^ = 94.87%		R^2^ (adj.) = 93.47%		R^2^ (pred.) = 90.19%	

**Table 7:** Results of the ANOVA of the revised Equation (8) for predicting E_prot_.

ANOVA F_critical_ (DF_regression_, DF_residual_, α) (9, 5, 0.05) = 4.77					
Source	Degree of Freedom (DF)	Adj. Sum of Square	Adj. Mean Square	F-Value	*p*-Value
Regression	9	1,103,426	122,603	4.34	0.060
Residual	5	141,283	28,257		
Lack of fit	3	136,363	45,454		
Pure error	2	4921	2460		
Total	14	1,244,710			
Coded Coefficient					
T_critical_ (DF_residual_, α) (5, 0.05) = 2.57					
Term	Coefficient	Standard Error Coefficient	T-value	*p*-value	Remark
Constant	260.7	97.1	2.69	0.044	<0.05
A	−12.4	59.4	−0.21	0.843	
B	147.8	59.4	2.49	0.055	
C	−287.9	59.4	−4.84	0.005	<0.05
AA	−102.7	87.5	−1.17	0.293	
BB	132	87.5	1.51	0.192	
CC	72.8	87.5	0.83	0.443	
AB	−15	84	−0.18	0.865	
AC	5.3	84	0.06	0.953	
BC	−182.5	84	−2.17	0.082	
Summary model					
R^2^ = 88.65%		R^2^ (adj.) = 68.22%		R^2^ (pred.) = 00.00%	

**Table 8:** Binding energies of the primary compounds of the passive film.

Element	Peak	Species (Binding Energy/eV)	Reference
Cr	2p3/2	Cr_met_ (573.6 ± 0.1); Cr_2_O_3_ (576.0 ± 0.1); Cr(OH)_3_ (577.3 ± 0.1)	[36]
Mo	3d5/2	Mo_met_ (227.3 ± 0.1); Mo^4+^ (228.5 ± 0.1); Mo^6+^ (231.8 ± 0.1)	[37,38,39]
	3d3/2	Mo_met_ (230.5 ± 0.1); Mo^4+^ (232.9 ± 0.1); Mo^6+^ (234.8 ± 0.1)	
Fe	2p3/2	Fe_met_ (706.5 ± 0.1); FeS_2_ (707.3 ± 0.1); Fe_3_O_4_ (709.5± 0.1); Fe_2_O_3_ (710.7 ± 0.1); FeOOH (711.8 ± 0.1); Fe_2_(SO_4_)_3_ (713.0 ± 0.1)	[15,40,41]
O	1s	O^2−^ (529.9 ± 0.1); OH- (531.2 ± 0.1); H_2_O (532.6 ± 0.1)	[42,43]
S	2s	S^2−^ (225.8 ± 0.1)	[44,45,46,47]
	2p3/2	S^2−^ (161.2 ± 0.1); S_2_^2−^ (162.8 ± 0.1); SO_4_^2−^ (168.3 ± 0.1)	
	2p1/2	S^2−^ (162.6 ± 0.1); S_2_^2−^ (164.8 ± 0.1); SO_4_^2−^ (169.6 ± 0.1)	
Ni	2p3/2	Ni_met_ (852.5 ± 0.1); NiO (853.7 ± 0.1)	[48]

**Table 9:** Area fraction ratio of deconvolted XPS spectra of passive film.

Concentration of HS^−^ (mM)	Area Fraction Ratio	
	Cr(OH)_3_/Cr_2_O_3_	OH^−^/O^2−^
0.01	0.70	0.91
1	0.79	1.08

## Data Availability

Data are contained within the article and supplementary materials.

## Supplementary Materials

The following supporting information can be downloaded at: https://www.mdpi.com/article/10.3390/ma17010178/s1, Figure S1: Surface OM images of specimens after experiments under all CCP conditions; Figure S2. Surface CLSM images of specimens after experiments under 0.001 M Cl^−^ CCP conditions (a) pH 8, 0.1 mM HS^−^, (b) pH 9, 0.01 mM HS^−^, (c) pH 9, 1 mM HS^−^, and (d) pH 10, 0.1 mM HS^−^; Table S1. Summary revised Models E_break_ of Model (5); Table S2. Summary revised Models of E_prot_.
