# Supplementary material for: Effect of Sulfide and Chloride Ions on Pitting Corrosion of Type 316 Austenitic Stainless Steel in Groundwater Conditions Using Response Surface Methodology
Source: Materials (Basel). 2023 Dec 28;17(1):178. doi: 10.3390/ma17010178 (PMC10779458; doi:10.3390/ma17010178)
Supplement: Supplementary file 1 [file materials-17-00178-s001.zip › materials-2785501-supplementary.pdf]

materials

Supplementary materials

Effect of Sulfide and Chloride Ions on Pitting Corrosion of Type 316 Austenitic Stainless Steel in Groundwater Conditions Using Response Surface Methodology

Jin-Seok Yoo †, Nguyen Thuy Chung †, Yun-Ho Lee, Yong-Won Kim and Jung-Gu Kim *

Department of Materials Science and Engineering, Sungkyunkwan University, 2066, Seobu-Ro, Jangan-Gu, Suwon-Si 16419, Republic of Korea; wlstjr5619@skku.edu (J.-S.Y.); chung.ngthuy@g.skku.edu (N.T.C.); yunho0228@naver.com (Y.-H.L.); dyddnjs98@skku.edu (Y.-W.K.)

* Correspondence: kimjg@skku.edu

† These authors contributed equally to this work.

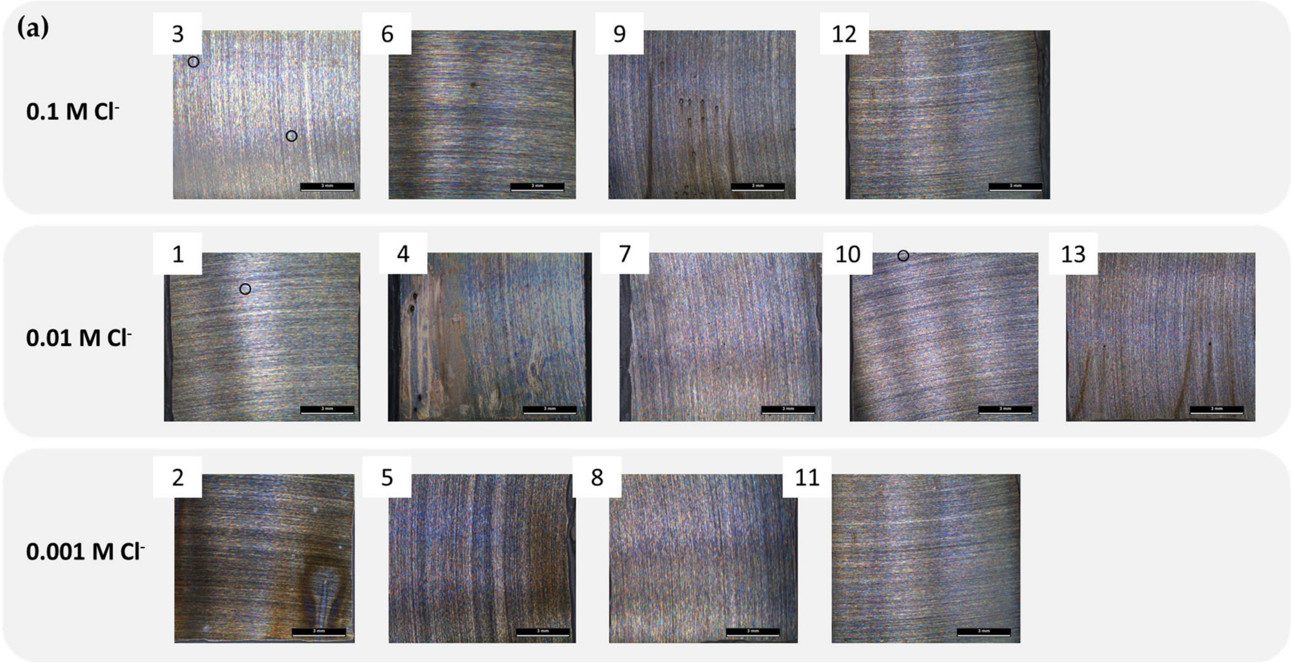

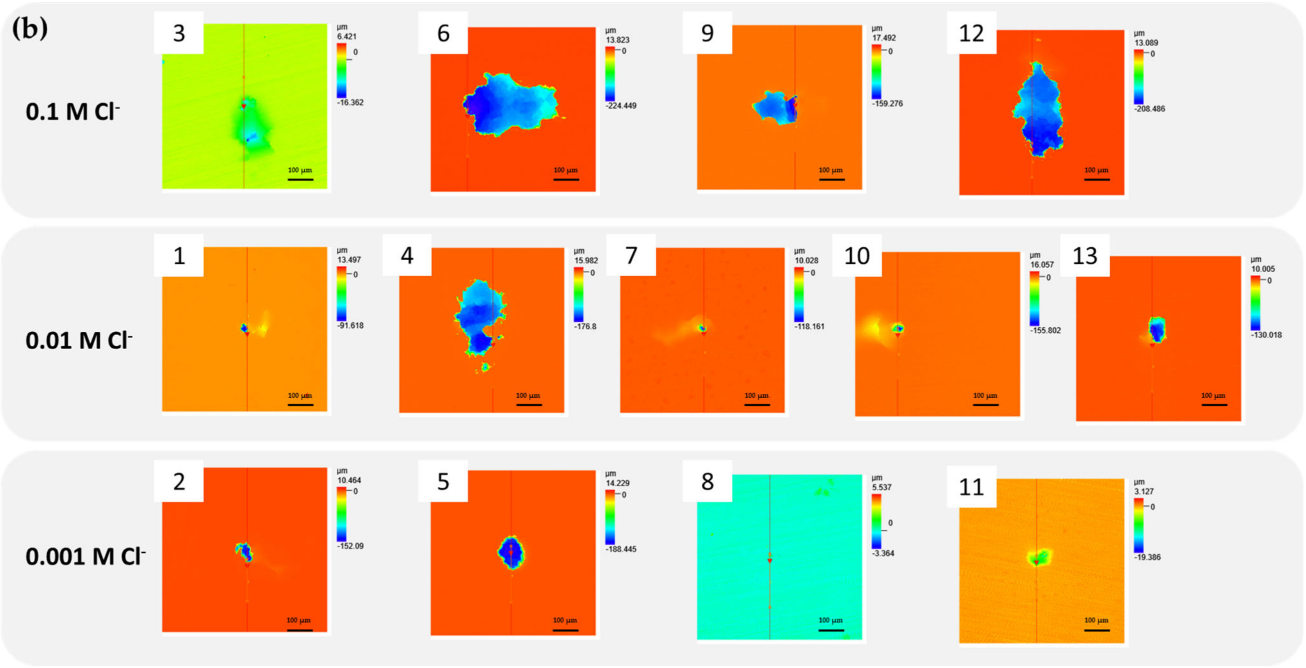

Figure S1. Surface images of specimens after experiments under all CCP conditions (a) OM and (b) CLSM.

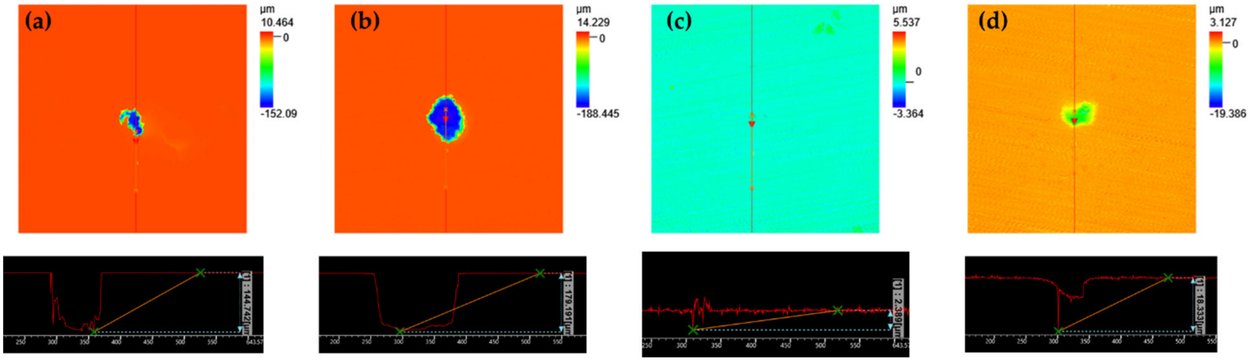

Figure S2. Surface CLSM images of specimens after experiments under 0.001 M Cl^- CCP conditions (a) pH 8, 0.1 mM HS^-, (b) pH 9, 0.01 mM HS^-, (c) pH 9, 1 mM HS^- ,and (d) pH 10, 0.1 mM HS^-

Table S1. Summary of revised Equations E_{break}

Equation	Terms	R^2 (%)	R^2 (adj.) (%)	R^2 (pred.) (%)
5	original	96.8	91.05	52.73
5.1	Eliminate AA	96.8	92.54	68.73
5.2	Eliminate AA + BC	96.74	93.47	73.23
5.3	Eliminate AA + BC + AC	96.64	94.11	79.12

5.4	Eliminate AA + BC + AC + CC	96.44	94.46	84.41
5.5(6)	Eliminate AA + BC + AC + CC+ A + AB	96.66	93.21	90.03
5.6	Eliminate AA + BC + AC + CC+ A + AB + B + BB	91.33	90.66	89.22

Table S2. Summary of revised Equations of E_{prot}

**Equation**	**Terms**	**R^2 (%)**	**R^2 (adj.) (%)**	**R^2 (pred.) (%)**
8	Original	88.65	68.22	0.00
8.1	Delete AC	88.64	73.49	0.00
8.2	Delete AC + AB	88.57	77.14	0.00
8.3	Delete AC + AB + A + AA	85.34	77.20	18.00
8.4	Delete AC + AB + A + AA + CC	83.40	76.76	28.73
8.5	Delete AC + AB + A + AA + CC + BB	78.00	72.00	18.85
8.6	Delete AC + AB + A + AA + CC + BB + BC	67.29	64.84	36.11
8.7	Delete AC + AB + A + AA + CC + BB + BC + B	53.26	49.67	29.21
